# MicroRNA Is Downregulated in Invasive Non-Functioning Pituitary Adenomas

**DOI:** 10.3390/ijms26094408

**Published:** 2025-05-06

**Authors:** Aleksandra Derwich-Rudowicz, Aleksandra Żbikowska, Marek Ruchała, Mirosław Andrusiewicz, Jakub Moskal, Nadia Sawicka-Gutaj

**Affiliations:** 1 Department of Endocrinology, Metabolism and Internal Medicine, Poznan University of Medical Sciences, Przybyszewskiego 49, 60-355 Poznań, Poland; aleksandra.derwich@student.ump.edu.pl (A.D.-R.);; 2 Doctoral School, Poznan University of Medical Sciences, Bukowska 70, 60-812 Poznań, Poland; 3 Department of Cell Biology, Poznan University of Medical Sciences, Rokietnicka 5D, 60-806 Poznań, Poland; 4 Department of Neurosurgery, Poznan University of Medical Science, Przybyszewskiego 49, 60-355 Poznań, Poland

**Keywords:** miRNA, pituitary tumor, invasive pituitary adenoma, prolactinoma

## Abstract

The study aimed to analyze hsa-miR-16-5p, hsa-miR-143-3p, hsa-miR-423-5p, hsa-miR-137-3p, hsa-miR-489-5p, hsa-miR-520-3p, hsa-miR-486-5p, and hsa-miR-200a-3p expression in the serum of patients with invasive non-functioning pituitary adenomas (NFPAs) and prolactinomas, as candidates for non-invasive biomarkers. The study included 62 patients with NFPAs and 18 with macroprolactinoma who qualified for transsphenoidal surgical resection. MicroRNAs were isolated from serum samples. The expression levels of hsa-miR-16-5p, hsa-miR-143-3p, hsa-miR-423-5p, hsa-miR-137-3p, hsa-miR-489-5p, hsa-miR-520-3p, hsa-miR-486-5p, and hsa-miR-200a-3p were determined using TaqMan MicroRNA assays. The statistical analyses were performed with MedCalc. The total concentration of microRNA was significantly lower in NFPAs than in the CG (*p* = 0.0419). ROC curve analysis showed that the cutoff point of miRNA lower than 10.73 predicted the PA (sensitivity = 70.0%; specificity = 57.7%; AUC = 0.629; *p* = 0.052). No correlation between selected miRNAs and tumor type was found: hsa-miR-143-3p (*p* = 0.4610), hsa-miR-16-5p (*p* = 0.8767), and hsa-miR-423-5p (*p* = 0.1459). miRNA expression also did not correlate with invasiveness (cavernous or sphenoid sinus invasion, optic chiasm compression). Although the total expression of microRNA was significantly lower in NFPAs, hsa-miR-16-5p, hsa-miR-143-3p, and hsa-miR-423-5p are not useful as non-invasive biomarkers in patients with invasive non-functioning pituitary adenomas and prolactinomas.

## 1. Introduction

Pituitary tumors (PTs) are a heterogeneous group of central nervous system lesions [1]. They are the second most common intracranial neoplasm, with the overall incidence estimated between 680 and 1430 cases per million people. Invasive adenomas account for 22–55% of pituitary adenoma (PA) cases, with invasiveness defined as the tumor invading surrounding structures, such as the cavernous sinuses and sphenoid sinus, as well as the focal or extensive bones [2]. Aggressive PAs, classified based on their invasiveness, rapid tumor growth, resistance to treatment, and multiple recurrences despite standard approaches, including surgical, pharmacological, and radiotherapy treatment [3], represent 2% of surgically resected tumors [4,5]. The prevalence of pituitary adenomas continues to rise, possibly due to improved radiographic imaging techniques. Their incidence varies according to age and gender but is slightly more common in women between 40 and 60 years. The most common pituitary tumors are prolactinomas (40–66%), followed by clinically non-functioning pituitary adenomas (NFPAs, 15–43%), somatotropinomas (8–16%), corticotropinomas (2–6%) and, rarely, thyrotropinomas (<1%) or gonadotropinomas [6]. Functioning pituitary tumors (hormone-producing) are usually diagnosed earlier than non-functioning pituitary tumors [7].

Surgical resection of the tumor mass is the first-line treatment for all hormonally active PTs, except prolactinomas [8]. However, surgery should be discussed alongside dopamine agonist treatment as a first-line option, especially in patients with rapidly progressive vision loss due to a sellar mass, as well as in younger patients, with a high chance of cure [9]. Patients with prolactinoma who do not respond to pharmacological treatment or experience severe side effects should also undergo surgical resection. Almost 90% of pituitary tumors can be safely excised using a transsphenoidal approach under fluoroscopic guidance and microsurgical techniques. Radiotherapy is usually used for residues or recurrences in inoperable sites [10,11]. The clinical outcomes of pituitary tumors highly differ; some remain stable for a long time, many grow slowly, and in rare cases, rapid tumor growth may also be observed [12]. After surgery, approximately 30% of patients show tumor regrowth, and the risk of tumor progression in the presence of a residual tumor is increased [13]. The treatment is challenging due to a lack of treatment targets and a limited understanding of the molecular mechanisms responsible for the development of these tumors [14]. The exact pathogenesis of pituitary adenomas is not well understood, but it is thought to be related to a combination of genetic and environmental factors. Almost 95% of all PAs are sporadic [5]. Although the genetic background remains unknown, the relevance of epigenetic changes is increasingly being raised. The pituitary epigenetic changes at the chromatin (pretranscription) and RNA levels (post-transcription) seem crucial in determining clinical characteristics, such as subtype differentiation and local invasion.

MicroRNAs (miRNAs) are small (about 19–25 nucleotides) non-coding RNA molecules involved in the post-transcriptional regulation of gene expression. They are a significant class of molecular regulators, regulating about 30% of human genes. They bind primarily to the 3′ untranslated regions and repress protein expression via the degradation of targeted mRNAs [15,16]. Since the first report, more than 1500 human miRNAs have been described. Recent studies have explored its use and ability to diagnose, prognose, and detect recurrence in many neoplasms, such as prostate cancer, breast cancer, ovarian cancer, colorectal cancer, and thyroid cancer [17,18]. In 2005, miRNAs were shown to be expressed in the pituitary gland [19]. They are also present in blood, saliva, cerebrospinal fluid, or follicular fluid of the ovary [20,21,22,23,24]. In fact, unlike cellular RNA, circulating miRNAs are stable in human body fluids and resistant to adverse conditions, such as acidic or alkaline pH, high temperature, and multiple freeze–thaw cycles [9]. Due to these characteristics and the fact that their expression level is closely related to pathology, miRNAs are candidates for new, non-invasive biomarkers. In pituitary adenomas, miRNA expression is primarily associated with PA tumorigenesis and treatment resistance, and the miRNAs with differential expression that have been identified in exosomes of PA patients suggest that miRNAs are selectively sorted into exosomes involved in tumor progression, invasion, and non-hormonal effects [14]. hsa-miR-16 was among the first identified miRNAs with a tumor-suppressive role in PAs [25]. Elevated expression of hsa-miR-200a was associated with PA invasion [26]. hsa-miR-143 might play a role in tumor progression or response to surgery [27]. hsa-miR-486-5p may serve as a significant prognostic marker in NFPAs, as patients with high hsa-miR-486 expression exhibited an increased risk of tumor relapse or residual progression [16]. In a comprehensive bioinformatic analysis aimed at identifying microRNAs associated with the aggressiveness of prolactinomas, hsa-miR-489 emerged as a potential biomarker for early diagnosis and personalized treatment of aggressive tumors [28]. hsa-miR-137 has been shown to inhibit cell survival, proliferation, and migration in both in vitro and in vivo models of prolactinoma [29]. Low expression of hsa-miR-137 is correlated with increased invasiveness and a higher frequency of tumor relapse [29].

Due to that knowledge, the study aimed to analyze hsa-miR-16-5p, hsa-miR-143-3p, hsa-miR-423-5p, hsa-miR-137-3p, hsa-miR-489-5p, hsa-miR-520-3p, hsa-miR-486-5p, and hsa-miR-200a-3p expression in the serum of patients with invasive non-functioning pituitary adenomas (NFPAs) and prolactinomas as candidates for non-invasive biomarkers.

## 2. Results

The analysis included 62 patients (32 females, 30 males) with non-functioning pituitary adenomas aged 20–83 and 18 patients with prolactinomas (four females, 14 males) aged 21–70 who qualified for tumor resection. The basic characteristics of the study groups are summarized in Table 1.

In the NFPA group, all patients presented with invasive tumors: 92% with macroadnomas and 8% with giant adenomas. Optic chiasm compression was present in 73% of the cases, followed by visual field defect in 63%. Concurrent hyperprolactinemia caused by pituitary stalk compression was found in 39% of cases. Hypopituitarism was present in over 50% of patients; the most common was the hypofunction of the gonadotropic axis (48%), followed by the thyreotropic (35%), corticotropic (32%), and somatotropic (6%) axes. Panhypopituitarism was present in six patients (10%).

Most patients in the PRL group were males (78%). Macroadenoma was present in 13 patients, and giant adenoma was present in five patients. The mean volume of the tumor was larger than in NFPAs; in 89%, there was an invasion of the cavernous and/or sphenoid sinus, and 72%. Optic chiasm compression was found in 72%, causing visual field defects in 50% of patients. The gonadotropic axis was impaired in 50% of the patients, followed by corticotropic (33%) and thyrotropic (33%) dysfunction. Panhypopituitarism was found in one patient.

Table 2 presents the laboratory results of all studied groups. The total concentration of microRNA was significantly lower in the NFPA group than in the CG (*p* = 0.0419). We did not observe that for prolactinoma. There was no significant difference in the mean concentration of hsa-miR-143-3p, hsa-miR-16-5p, or hsa-miR-423-3p in PAs in comparison to healthy controls (*p* = 0.568, *p* = 0.107, *p* = 0.523, respectively). We were not able to target targeted hsa-miR-137-3p, hsa-miR-489-5p, hsa-miR-520-3p, hsa-miR-486-5p and hsa-miR-200a-3p, and we excluded them from further analyses. We observed significantly lower levels of follicle-stimulating hormone (FSH), luteinizing hormone (LH), estradiol, testosterone, and fT4 in NFPAs and prolactinomas than in the control group (*p* < 0.05). Insulin-like growth factor 1 (IGF-1) and free triiodothyronine (fT3) were significantly lower in the NFPAs group than the CG (*p* = 0.024 and *p* = 0.0067, respectively).

**Figure 1:**
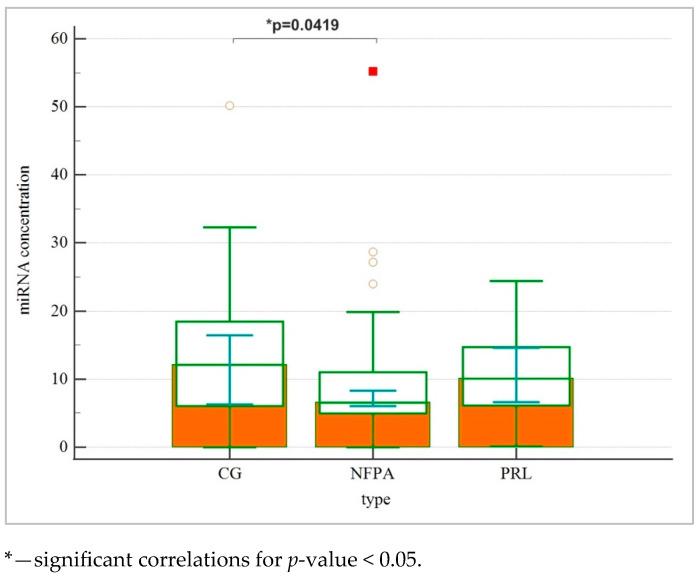
Multiple comparison graph of miRNA concentration. The miRNA concentration was significantly lower in the NFPA group than in the CG (*p* = 0.0419). We did not observe that for prolactinoma.

**Figure 2:**
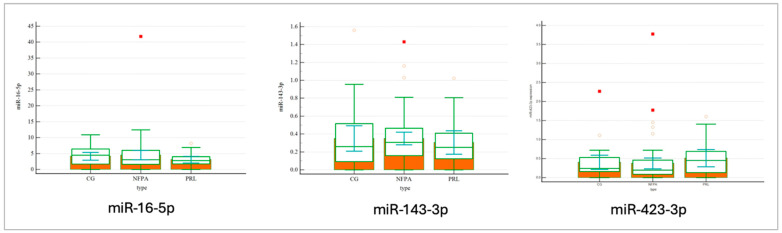
No differences between selected miRNAs and tumor type were found: hsa-miR-143-3p (*p* = 0.4610), hsa-miR-16-5p (*p* = 0.8767), hsa-miR-423-5p (*p* = 0.1459).

Table 3 shows correlations between hsa-miR-16-5p, hsa-miR-143-3p, hsa-miR-423-3p and total miRNA concentration in patients with NFPAs, prolactinomas and healthy controls. The total concentration of microRNA differed according to tumor type (*p* = 0.0083). The Kruskal–Wallis test revealed that miRNA concentration was significantly lower in the NFPA group than in the CG (*p* = 0.0419) (Figure 1). We did not observe that for prolactinoma. No correlations between selected miRNAs and tumor type were found, neither for miR-143-3p (*p* = 0.4610), nor hsa-miR-16-5p (*p* = 0.8767), or hsa-miR-423-5p (*p* = 0.1459) (Figure 2).

In the multiple regression analysis, we confirmed the association between microRNA and tumor type (coefficient = 2.5451; *p* = 0.0157), whereas age and sex did not contribute significantly. The data are presented in Table 4a,b.

The miRNA expression did not correlate with tumor volume, patient’s age, invasion, presence of tumor mass effect symptoms, or body parameters (Table 5). We found a weak inverse relationship between miRNA concentration and sphenoid sinus invasion (Rs = −0.231; *p* = 0.0390).

**Figure 3:**
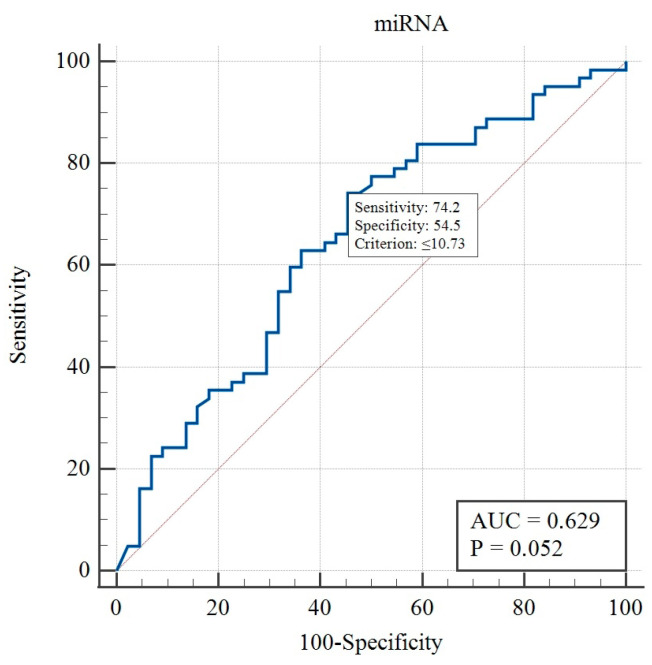
ROC curve analysis for miRNA concentration.

ROC curve analysis showed that the cutoff point of miRNA lower than 10.73 predicted the PA (sensitivity = 70.0%; specificity = 57.7%; AUC = 0.629; *p* = 0.052, Figure 3).

## 3. Discussion

Our study analyzed miRNA expression in the serum of patients with invasive, non-functioning pituitary adenomas and prolactinomas. All patients were assessed at the time of diagnosis. We found that the total concentration of microRNA differed according to tumor type and was significantly lower in the NFPA than in the control group. We analyzed the expression of hsa-miR-143-3p, hsa-miR-16-5p, and hsa-miR-423-3p in serum, but we did not observe any significant correlation between selected miRNAs and tumor type. We can conclude that hsa-miR-143-3p, hsa-miR-16-5p, and hsa-miR-423-5p cannot be used as non-invasive biomarkers for NFPAs or prolactinomas. However, knowing that total miRNA expression is downregulated, further studies investigating these underlying molecular mechanisms and potential biomarkers are urgently needed. We also targeted hsa-miR-137-3p, hsa-miR-489-5p, hsa-miR-520-3p, hsa-miR-486-5p, and hsa-miR-200a-3p. However, they were excluded from further analysis because we did not obtain any expression in PCR. Probably, miRNA expression was at such a low level that we could not detect it.

Our finding of a lower total concentration of microRNA in the NFPA group than in the control group was consistent with the study by Németh et al. [23], which revealed a global downregulation of miRNA expression level in plasma samples from patients with pituitary adenomas compared with samples obtained from healthy controls. This data suggest that miRNA plays an important role in pituitary tumorigenesis and cell cycle regulation. ROC curve analysis showed that the cutoff point of total miRNA concentration lower than 10.73 might predict the PA; however, the sensitivity and specificity of this test are not satisfactory enough.

NGS analysis of exosomal miRNA detected only 13.6% of known miRNAs in high expression levels, while all novel miRNAs were in middle or low expression levels [16]. In blood samples, the expression differed between patients with NFPAs and healthy controls, and a total of 54 mature miRNAs showed significant alteration in expression, which included 18 up-regulated and 36 down-regulated miRNAs in the NFPA group [16].

Zhang et al. [30] found that hsa-miR-143 was significantly downregulated in pituitary tumor tissues compared to noncancerous controls. Also, hsa-miR-143 expression in three pituitary tumor cell lines (GH3, MMQ, and AtT-2) was downregulated. miR-143 not only mimicked inhibition of pituitary tumor cell proliferation and promoted apoptosis in GH3 and MMQ cell lines but also acted as a tumor suppressor and directly targeted oncogene K-Ras. Hsa-miR-143-3p was also significantly overexpressed in preoperative FSH/LH plasma samples compared with their 3-month–postoperative pairs and preoperative hormone-immunonegative samples [27]. The downregulation of hsa-miR-143-3p has also been observed in ovarian, gastric, pancreatic, and prostate cancer, which speaks for the possibility of its tumor suppressor nature [31,32,33,34,35]. Unfortunately, our study did not confirm that thesis.

In a study using a serum exosomal miRNA profile-based method to screen NFPAs and predict prognosis, a total of 1395 human miRNAs were detected by NGS. Compared with healthy controls, 18 upregulated and 36 downregulated miRNAs showed significant expression changes in NFPA patients, with hsa-miR-486-5p, hsa-miR-151a-5p, hsa-miR-652-3p_R + 1, and hsa-miR-1180-3p identified as promising biomarkers. miR-486-5p was the most accurate and efficient biomarker for predicting progression and relapse among NFPA patients [16]. We also targeted miR-486-5p, but, as mentioned before, it was excluded from the analysis due to the lack of expression detected in PCR.

A few different studies revealed that hsa-miR-16 is deregulated in PAs and associated with the occurrence of cancer and poor prognosis [19,36,37]. Bottoni et al. [19] showed that the expression level of miR-16 is significantly decreased in PA tissues compared with normal pituitary tissues. Its downregulation correlated with a greater tumor diameter and a lower p43 secretion. Renjie and Haiqian [36] found that miR-16 was downregulated in invasive pituitary tumor cells and tissues, and it suppressed the cell proliferation, migration, and invasion. Moreover, the expression level of miR-16 was significantly increased in patients with invasive PAs compared to patients with non-invasive PAs. Niu et al. [37] demonstrated that the expression of miR-16 was significantly decreased in patients with PAs compared to normal pituitary tissue, whereas the expression of HMGA2 mRNA was significantly increased. In cell culture, the transfection of HP75 cells with siRNAs targeting HMGA2 and/or miR-16 mimics suppressed HMGA2 expression, decreased the cells’ proliferative ability, and promoted apoptosis. This led to the hypothesis that miR-16 inhibited the proliferation and promoted apoptosis of HP75 cells by inhibiting HMGA2 expression [37]. Amaral et al. [38] analyzed the differential expression of let-7a, miR-15a, miR-16, miR-21, miR-141, miR-143, miR-145, and miR-150 in corticotropinomas and revealed their underexpression compared with normal pituitary tissues. Unlike Bottoni et al. [19], they found no differences between miRNA expression and tumor size. Studies on plasma miRNAs circulating in PA patients are still limited. Despite the results confirming altered miRNA expression in pituitary adenoma tissues, studies on circulating plasma miRNAs in PA patients often stand opposite and do not support the presence of these changes. In our study, we did not confirm the changed expression of this miRNA in plasma.

Another tissue study found the expression of hsa-miR-423-5p lower in somatotroph adenoma tissue than in healthy controls [39]. In vitro experiments showed that miR-423-5p inhibited cell proliferation, induced cell apoptosis, and reduced growth hormone release and migration of GH3 cells [39]. It leads to the conclusion that hsa-miR-423-5p is important in promoting tumorigenesis and cancer growth in PAs. Hsa-miR-423-5p was also found to be a potential biomarker for nasopharyngeal cancer and a tumor growth promotor in gastric cancer and hepatocellular cancer [39,40].

An increasing number of studies try to reveal the relationship between miRNA expression and the exact pathomechanism of pituitary tumors. There are reports about correlations between miRNA dysregulation in the peripheral blood and pathophysiological conditions [27,41]. However, much information is still missing, and the molecular mechanisms of PA tumorigenesis are not fully understood. Several limitations of our study need to be mentioned. Quantitative real-time polymerase chain reaction is still considered the gold standard for measuring miRNA expression, but it has limitations. We were not able to target targeted hsa-miR-137-3p, hsa-miR-489-5p, hsa-miR-520-3p, hsa-miR-486-5p and hsa-miR-200a-3p. Next-generation sequencing is the most reliable method for identifying and profiling miRNAs, and it allows for the detection of miRNA isoforms. Additionally, comparing miRNA expression in patients’ plasma with PA tissue would be valuable. Another factor may be the low number of healthy controls, who were not matched for sex and age. However, in multiple regression analysis, we excluded the effect of age and gender and confirmed the significance of the observed results.

Our results confirmed a lower level of circulating miRNAs in the plasma of patients with clinically significant non-functioning pituitary adenomas compared to healthy individuals and support the thesis that altered miRNA expression profile is involved in PA tumorigenesis.

## 4. Materials and Methods

### 4.1. Patients

A prospective study with consecutive enrollment was conducted [42]. The study included 62 patients with non-functioning pituitary adenomas (NFPAs) and 18 patients with macroprolactinoma hospitalized in the Department of Endocrinology, Metabolism and Internal Medicine of Poznan University of Medical Sciences in Poland between January 2022 and June 2024 and qualified for transsphenoidal surgical resection. Inclusion criteria were as follows: (1) patients with a clinically significant pituitary tumor (non-functioning pituitary adenoma (NFPA) or prolactinoma); (2) age above 18 years of age; and (3) giving informed written consent to participate in the study. Exclusion criteria included autoimmunological disorders, malignancy, pregnancy, and lack of written consent. A comprehensive medical interview was obtained, and age, gender, and BMI were reviewed.

The control group consisted of 26 healthy volunteers. Inclusion criteria were (1) normal pituitary gland on MRI; (2) absence of comorbidities; (3) age above 18; and (4) giving informed written consent to participate in the study.

This study was conducted in accordance with the Declaration of Helsinki and approved by the Bioethical Committee of Poznan University of Medical Sciences (Decision No. 207/22, 862/23). Patients were informed of the study aim and collection strategies, and each patient signed an informed consent. Patients were informed that they could withdraw from the study at any point. All methods adhered to relevant guidelines and regulations [43].

### 4.2. Hormone Level Assessment

Laboratory parameters included adrenocorticotropic hormone (ACTH), growth hormone (GH), insulin growth like factor-1 (IGF-1), luteinizing hormone (LH), follicle-stimulating hormone (FSH), thyroid-stimulating hormone (TSH), free triiodothyronine (fT3), free thyroxine (fT4), cortisol, testosterone, estradiol, prolactin, and sex hormone binding globulin (SHBG). Blood samples were collected after an overnight fast for the measurement of all parameters.

The assays were performed according to the manufacturer’s recommendations. ACTH, LH, FSH, SHBG, TSH, fT3, fT4, prolactin, and cortisol were measured with electrochemiluminescence (ECLIA) using the Cobas e801 analyzer (Roche Diagnostics, Indianapolis, IN, USA). GH levels were determined using ECLIA on the Cobas e402 analyzer (Roche Diagnostics, Indianapolis, IN, USA). IGF-1 levels were measured with the chemiluminescence (CMIA) method using a LIAISON Analyzer (DiaSorin Ltd., Saluggia, Italy).

### 4.3. Magnetic Resonance Imaging

All patients underwent pituitary gland MRI scans to assess image characteristics, including tumor size, intratumor hemorrhage, and invasion type. MRI was performed using Siemens Magnetom Skyra (serial number 145114). Tumor size was determined by measuring the greatest diameter, with macroadenoma defined as a tumor with a diameter of ≥10 mm. The invasion type was determined based on the invasion site of the tumor and recorded as cavernous sinus invasion, sphenoid sinus invasion, or suprasellar invasion. The diagnosis was confirmed by postoperative pathology, and immunohistochemical examination was used to determine the endocrine type in accordance with the WHO classification of endocrine system tumors.

### 4.4. MicroRNA Serum Level Assessment

**RNA processing and extraction for genetic analysis.** Peripheral blood (5 mL) was collected during routine medical procedures into tubes containing the clot activator S-Monovette^®^ Serum, CAT (Sarstedt, Nümbrecht, Germany) and then centrifuged at 2000× *g* for 20 min at 4 °C to separate the serum. Collected serum samples were stored at −80 °C until nucleic acid extraction. MicroRNAs, without the high molecular RNA fraction, were isolated from serum samples using the double-column system for miRNA and RNA isolation, according to the manufacturer’s protocol (A&A Biotechnology, Gdansk, Poland) and eluted in 100 µL of ultra-pure water. Isolated miRNA fractions were stored at −80 °C. The quantity and purity of isolated miRNAs were measured spectrophotometrically.

**Complementary miRNA DNA (cmiDNA) synthesis**. The cmiDNA was synthesized in a four-step reaction with the TaqMan Advanced miRNA cDNA Synthesis Kit (Thermo Fisher Scientific, Waltham, MA, USA), using two nanograms of miRNA. The cmiDNAs were prepared in duplicate for each sample and used as qPCR templates.

**Figure 4:**
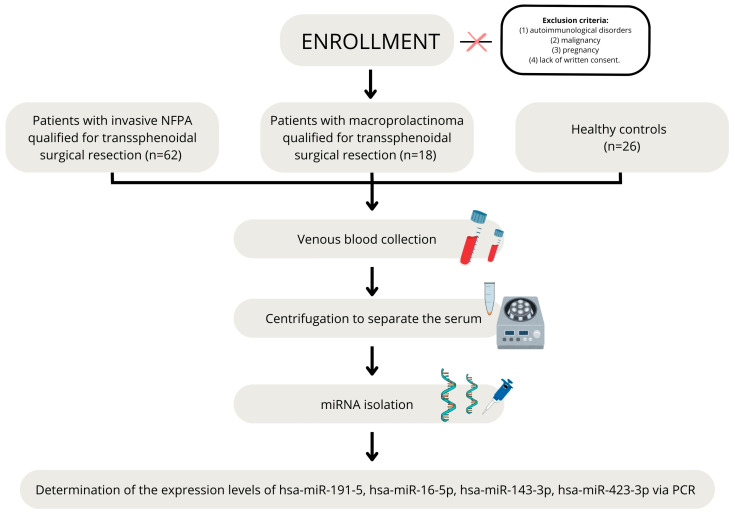
Study flow chart.

**Quantitative polymerase chain reaction (qPCR)**. The qPCR was performed using SolisFAST Probe qPCR Mix with UNG (Solis Biodyne, Tartu, Estonia) according to the manufacturer’s protocol in a total volume of 20 µL. The expression levels of hsa-miR-191-5p (assay ID: 477952_mir), hsa-miR-16-5p (assay ID: 477860_mir), hsa-miR-143-3p (assay ID: 477912_mir), hsa-miR-423-3p (assay ID:478090_mir), hsa-miR-137-3p (assay ID: 477904_mir), hsa-miR-489-5p (assay ID: 478940_mir), hsa-miR-520-3p (assay ID: 479509_mir), hsa-miR-486-5p (assay ID: MC10546), and hsa-miR-200a-3p (assay ID: 478490_mir) were determined using TaqMan MicroRNA Assays (Thermo Fisher Scientific, Waltham, MA, USA). The hsa-miR-191-5p served as a reference miRNA [44]. The thermal profile and acquisition steps were performed per the SolisFast master mix protocol. Each miRNA sample and reference were reaction efficiency corrected. Cobas Z480 analyzer (Roche Diagnostics, Basel, Switzerland) and the dedicated software LCS480 1.5.1.62 SP3–UDF 2.1.0.26 (Roche Diagnostics, Basel, Switzerland) were used for the qPCR analyses. Relative expression level analysis was performed using the same software by comparing the expression level of the genes of interest with that of the reference gene. Figure 4 presents study flow chart.

### 4.5. Statistical Analyses

Statistical analysis was performed with MedCalc Statistical Software version 19.1.5 (MedCalc Software bv, Ostend, Belgium). Normality was analyzed by the D’Agostino-Pearson test. A comparison of results between the study and control groups was performed using the ANOVA and Kruskal–Wallis tests. A paired samples *t*-test was used to compare and analyze parameters in normally distributed data. Spearman’s rank correlation coefficient was used to find relationships between the analyzed parameters. Receiver operating characteristic (ROC) curves were calculated to determine the potential of miRNA concentration to discriminate between patients with PAs and healthy controls. An optimal cutoff point was calculated according to the highest accuracy (minimal false-negative and false-positive results). The area under the ROC curve (AUC) was used to check the prognostic value of a particular parameter. Simple regression analysis was used to test for the relationships between miRNA and tumor type, age, and gender. Furthermore, stepwise multiple regression analysis was employed to investigate the influence of various parameters on miRNA. Variables were entered into the model if their associated *p*-values were less than 0.05 and then sequentially removed if their associated *p*-values became greater than 0.2. The *p*-value of less than 0.05 was considered statistically significant.

## Figures and Tables

**Table 1:** Basic characteristics of the studied groups.

	NFPA (*n* = 62)	PRL (*n* = 18)	CG (*n* = 26)
Age (M ± SD)	57.3 ± 15.6	42.5 ± 14.0	42.2 ± 13.1
Gender (males/females)	30 (48%)/32 (52%)	14 (78%)/4 (22%)	9 (35%)/17 (65%)
Macroadenoma	57 (92%)	13 (72%)	NA
Giant adenoma	5 (8%)	5 (28%)	NA
Volume (cm^3^) (M ± SD)	7.95 (±7.08)	10.49 (±13)	NA
Invasive/Non-invasive	62 (100%)/0	16 (89%)/2 (11%)	NA
Invasion of cavernous sinus	57 (92%)	14 (78%)	NA
Invasion of sphenoid sinus	11 (18%)	2 (11%)	NA
Optic chiasm compression	45 (73%)	13 (72%)	NA
Tumor mass effect			
Headache	54 (87%)	11 (61%)	NA
Visual field defect	39 (63%)	9 (50%)	NA
Hyperprolactinemia	24 (39%)	18 (100%)	NA
Hypopituitarism			
Gonadal axis	30 (48%)	9 (50%)	NA
Corticotropic axis	20 (32%)	6 (33%)	NA
Somatotropic axis	6 (10%)	1 (6%)	NA
Tyreotropic axis	22 (35%)	6 (33%)	NA

NFPA—non-functioning pituitary adenoma; PRL—prolactinoma; CG—control group; NA—not applicable.

**Table 2:** Laboratory results of all studied groups.

Median (95% CI)	NFPA	PRL	CG	*p*-Value
ACTH [pg/mL]	29.8 (23.6–35.4)	39.6 (26.4–45.6) *	18.9 (13.7–22.2)	0.006
GH [ng/mL]	0.26 (0.17–0.46)	0.24 (0.07–0.49) *	0.89 (0.35–1.95)	0.018
Cortisol [nmol/L]	339 (271–408)	331 (273–423)	395 (340–471)	0.066
TSH [μU/mL]	1.10 (0.90–1.47) *	1.89 (1.02–2.22)	1.77 (1.28–2.41)	0.003
fT3 [pmol/L]	3.9 (3.7–4.1) *	4.0 (3.4–4.4)	4.5 (4.2–4.8)	0.006
fT4 [pmol/L]	14.1 (13.4–15.0) *	12.5 (9.3–15.0) *	15.5 (14.6–16.2)	0.002
IGF1 [ng/mL]	M 101 (76–153) * F 154 (81–191)	M 106 (69–149) * F 195 (130–263)	M 174 (120–205) F 161 (110–204)	M 0.0036 F 0.39
PRL [μIU/mL]	302 (234–403)	4220 (982–16697) *	249 (197–363)	0.000003
FSH [mlU/mL]	5.9 (4.5–7.8)	2.6 (1.5–3.6) *	5.9 (4.7–11.2)	0.002
LH [mlU/mL]	3.7 (2.5–6.1) *	2.1 (1.4–3.1) *	8.2 (4.8–14.1)	0.0008
Testosterone [nmol/L]	M 7.06 (2.12–10.7) * F 0.4 (0.1–0.8) *	M 4.93 (1.50–9.95) * F 1.13 (0.85–1.38)	M 19.2 (16.6–24.1) F 1.0 (0.85–1.3)	M 0.0067 F 0.0005
SHBG [nmol/L]	M 30.9 (25.4–63.2) F 63.8 (46.2–83.8)	M 38.3 (26.8–41.2) F 63.1 (49.5–79.8)	M 47.8 (36.8–52.6) F 67.3 (49.4–79.0)	M 0.19 F 0.99
Estradiol [pg/mL]	M 14 (4–20) F 7 (5–18)	M 18 (7–28) F 41 (23–145)	M 21 (19–27) F 40 (13–87)	M 0.055 F 0.057
miRNA concentracion	6.55 (6.06–8.35) *	10.08 (6.63–14.59)	12.11 (6.28–16.45)	0.031
hsa-miR-143-3p	28.72 (28.05–29.01)	29.08 (26.72–30.74)	29.67 (27.49–30.49)	0.568
hsa-miR-16-5p	24.22 (23.49–25.04)	25.26 (22.50–27.06)	25.38 (24.07–27.36)	0.107
hsa-miR-423-3p	28.42 (27.07–29.62)	27.74 (25.56–29.73)	28.84 (27.97–29.66)	0.523
hsa-miR-191-5p	28.05 (27.53–28.21)	27.97 (26.21–31.04)	28.92 (27.73–30.70)	0.348

NFPA—non-functioning pituitary adenoma; PRL—prolactinoma; CG—control group; LH—luteinizing hormone; FSH—follicle-stimulating hormone; PRL—prolactin; SHBG—sex hormone binding globulin; TSH—thyroid-stimulating hormone; fT3- free triiodothyronine; fT4—free thyroxine; GH—growth hormone; IGF1—insulin-like growth factor 1; ACTH—adrenocorticotropic hormone; M—males; F—females; *—significant difference between study group and CG; CI—confidence intervals.

**Table 3:** Correlations between hsa-miR-16-5p, hsa-miR-143-3p, hsa-miR-423-3p, and total miRNA concentration in patients with NFPAs, prolactinoma, and healthy controls (Spearman’s rank correlation coefficients).

	hsa-miR-143-3p		hsa-miR-16-5p		hsa-miR-423-3p		Total miRNA Concentration	
	Rs	*p*-Value	Rs	*p*-Value	Rs	*p*-Value	Rs	*p*-Value
Tumor type	–0.0724	0.461	0.0153	0.8767	0.142	0.1459	0.255	0.0083 *

Rs—Spearman’s rank correlation coefficients; *—significant correlations for *p*-value < 0.05.

**Table 4:** (**a**). Multiple regression analysis for total miRNA concentration (the models include tumor type and age). (**b**). Multiple regression analysis for total miRNA concentration (the model includes tumor type and gender).

(a)							
Independent Variables	Coefficient	Std. Error	95% CI	t	*p*-Value	r_partial_	r_semipartial_
(Constant)	6.6689	1.9316	2.8386 to 10.4993	3.4526	0.0008		
Type	2.5451	1.0367	0.4894 to 4.6009	2.4551	0.0157 *	0.2341	0.2341
(**b**)							
**Independent Variables**	**Coefficient**	**Std. Error**	**95% CI**	**t**	***p*-Value**	**r_partial_**	**r_semipartial_**
(Constant)	6.6689	1.9316	2.8386 to 10.4993	3.4526	0.0008		
Type	2.5451	1.0367	0.4894 to 4.6009	2.4551	0.0157 *	0.2341	0.2341

Std. Error—standard error; CI—confidence intervals; *—significant correlations for *p*-value < 0.05.

**Table 5:** Correlations between total miRNA concentration and patients’ characteristics (Spearman’s rank correlation coefficients).

	**Age**		**BMI**		**Body Weight**		**Headache**	
	Rs	*p*-Value	Rs	*p*-Value	Rs	*p*-Value	Rs	*p*-Value
miRNA concentration	–0.15	0.1326	–0.12	0.24	–0.01	0.8983	–0.16	0.165
	**Tumor volume**		**Visual field loss**		**Invasion of cavernous sinus**		**Invasion of sphenoid sinus**	
	Rs	*p*-value	Rs	*p*-value	Rs	*p*-value	Rs	*p*-value
miRNA concentration	<0.01	0.9915	0.07	0.5235	–0.23	0.0390 *	−0.10	0.368

Rs—Spearman’s rank correlation coefficients; *—significant differences for *p*-value < 0.05.

## Data Availability

The raw data supporting the conclusions of this article will be made available by the authors upon request.
